# Autism severity and its relationship to disability

**DOI:** 10.1002/aur.2898

**Published:** 2023-02-14

**Authors:** Einat Waizbard-Bartov, Deborah Fein, Catherine Lord, David G. Amaral

**Affiliations:** 1 Department of Psychology, University of California Davis, Davis, California, USA; 2 The MIND Institute and Department of Psychiatry and Behavioral Sciences, University of California Davis, Davis, California, USA; 3 Department of Psychological Sciences, University of Connecticut, Storrs, Connecticut, USA; 4 Department of Pediatrics, University of Connecticut, Farmington, Connecticut, USA; 5 Departments of Psychiatry and Human Development and Psychology, University of California, Los Angeles, California, USA

**Keywords:** ADOS calibrated severity score, autism severity, autism spectrum disorder, autism symptoms, profound autism, symptom severity change

## Abstract

Autism severity is currently defined and measured based exclusively on the severity levels of the two core symptom domains: social-communication and restricted or repetitive patterns of behaviors and interests. Autistic individuals, however, are often diagnosed with other medical, developmental, and psychological co-occurring conditions. These additional challenges such as intellectual disability, limited expressive and/or receptive language, and anxiety disorders, can have a tremendous impact on the day-to-day lives of autistic individuals, for both their adaptive functioning as well as their sense of wellbeing. Furthermore, the initial presentation of core symptoms and their likelihood of changing over time are influenced by the presence of such co-occurring conditions. In order to truly understand how a person’s autism impacts their life, both core symptoms as well as other challenges should be considered. This approach was recently taken by*The Lancet Commission on the future of care and clinical research in autism*, which proposed the term “profound autism” for a subgroup of individuals presenting with high core symptom severity, co-occurring intellectual disability, and little or no language, who require extensive long-term care. Considering other individual factors such as daily living skills, specific support needs and environmental resources would also enhance the evaluation of disability in autistic individuals. As currently employed in the assessment of intellectual disability, a multidimensional approach to autism could provide a more comprehensive system for classification of impairment. At present, however, there is no formal way to designate the combined effect of these different aspects of autism on a person’s life. A comprehensive outlook that acknowledges impairments, capabilities, co-occurring conditions, and environmental factors would be useful for identifying subgroups of individuals as well as for determining individual needs and strengths in clinical assessments.

## Lay Summary

The severity of a person’s autism is currently defined based on the severity of their core autism symptoms: impaired social-communication and the presence of restricted or repetitive patterns of behaviors and interests. But autistic people often face additional challenges such as intellectual disability, epilepsy, and anxiety disorder, that considerably impact their everyday life, wellbeing, and the need for support. A more complete view of autism severity, one that includes core symptoms as well as additional challenges, could help identify meaningful subgroups of autistic individuals and could be useful in clinical care.

## INTRODUCTION

Autism has historically been considered an impairing condition. Based on current DSM5 criteria (APA, 2013), autism includes three levels of severity ranging from “requiring support” to “requiring very substantial support.” Judgments of severity are based solely on the characteristics of the two core domains that make up the diagnostic criteria. The first domain comprises deficits in social and communication abilities (social affect). These can manifest, for instance, in failure to use eye contact to initiate communication with others, as well as in social interactions that lack a natural, “back and forth” reciprocal quality. The second core domain is the presence of restricted or repetitive patterns of behavior and interests (RRBs). These can range from engaging in repetitive movements, such as hand flapping, to having an intense preoccupation with highly specific objects or topics. The underlying assumption is that the way neurotypical individuals seek out, initiate and maintain social interactions represents a basic human behavior, and lack of interest or ability to participate in this behavior is considered to be a disability. Moreover, the presence of circumscribed or repetitive behaviors can limit an autistic individual’s experience of the varied human activities and is again, therefore, considered to be a disability. While clinicians also specify if there is accompanying intellectual or language impairment, these features are not commonly integrated into the overall judgment of autism severity. Moreover, other common, co-occurring conditions in autism such as sleep difficulties, gastrointestinal distress and epilepsy are also not usually considered in evaluating the level of autism severity. This is due to the fact that, as stated above, the DSM-5 specifies levels of autism severity based solely on the core symptom domains and three levels of needed supports, regardless of other individual characteristics.

The severity levels of social affect and repetitive behaviors are described with some specificity in DSM-5. For example, level 3 social communication entails “very limited initiation of social interactions, and minimal response to social overtures from others” while level 1 social communication entails “difficulty initiating social interactions, and clear examples of atypical or unsuccessful response to social overtures of others.” Furthermore, these severity levels are explicitly posited to correspond to different levels of functional impairment: Level 1, “without supports in place, deficits in social communication cause noticeable impairments”; Level 2 “social impairments apparent even with supports in place”; and Level 3, “severe deficits in verbal and nonverbal social communication skills cause severe impairments in functioning.” Thus, the DSM-5 attaches to the severity levels specific behaviors, degrees of impairment and required support resulting *only from autism-specific symptoms* rather than associated conditions.

Writing a commentary on autism severity at this time is fraught with potential controversy. The autism community is becoming increasingly polarized with some seeing little or no “disability” associated with autism (Kapp et al., 2013) while others view limited social ability as disabling in itself, and/or highlight the significant associated medical issues that cause substantial disability and limit daily functioning (Singer, 2022, November 11). This diversity of opinion on the level of disability associated with autism results, in part, from the enormous heterogeneity in how the core and co-occurring conditions present in individual autistic persons. It goes beyond this, however, since the level of disability is often moderated by environmental factors such as amount and efficacy of intervention, socioeconomic level and availability of quality support services.

In this commentary, we will first review how autism severity is evaluated in a research context. We will then briefly summarize evidence that autism severity in an individual is changeable during development and mention some of the factors that may be associated with severity changes. We conclude with a discussion of why a research estimation of autism severity that corresponds to the DSM-5 levels is not sufficient to closely map on to an individual’s real-life functioning, including their experiences and challenges. We describe the evolution of classification systems in intellectual disability that now use criteria that go well beyond IQ. In autism, we favor the use of such multidimensional classification systems to determine not only severity levels and concomitant impairment, but also to lead to an understanding of the individual based on his or her support needs. Such a system is consistent with the use of terms such as “profound autism” for those individuals who have not only severe autism symptoms per se but also significant challenges beyond the core symptoms of autism, such as intellectual disability. A multidimensional approach to defining autistic subgroups would undoubtedly achieve a more balanced picture of the impairments and capabilities which are both features of the autistic individual and promote better clinical characterization. It would probably also promote fundamental biological research. As pointed out by Waterhouse and Gillberg (2014), studying autism spectrum disorder (ASD) as a single syndrome and attempting to find a single underlying brain dysfunction has led research away from directions that may be more productive, specifically individual variation and existence of micro-subgroups.

## HOW IS AUTISM SEVERITY ASSESSED IN A RESEARCH CONTEXT?

Autism assessment in a research context is done in diagnostic settings and includes standard assessment tools for evaluation of core symptoms. There are different standardized tools available for this purpose, such as the Childhood Autism Rating Scale (CARS) (Schopler et al., 1986), the Autism Behavior Checklist (ABC) (Krug et al., 1980) and others. However, the gold standard measures for evaluation of autism symptoms are the Autism Diagnostic Interview-Revised (ADI-R) and the Autism Diagnostic Observation Schedule (ADOS). The ADI-R (Lord et al., 1994) is an in-depth clinical interview conducted with caregivers regarding the individuals’ current and past behavior. The ADOS (Lord et al., 2000) is a relatively short clinical observation in which a clinician directly assesses the presence of autism symptoms in the individual’s behavior. Five versions, geared to the individual’s age and level of functioning, provide appropriate standardized social probes. Each individual receives a version of the ADOS that is adapted to their language ability. In addition to assessing the presence of symptoms, these measures also produce evaluations of the severity levels of the core symptoms. The ADOS specifically includes for this purpose a standardized, 10-point severity metric known as the Calibrated Severity Score (CSS) (Esler et al., 2015; Gotham et al., 2009; Hus & Lord, 2014). The CSS yields a symptom severity rating that is standardized relative to individuals of the same age and language abilities. The strength of this approach is that it deliberately limits the impact of other characteristics when evaluating symptom presentation, allowing a non-biased assessment of autism core symptoms.

## WHAT IS THE EVIDENCE THAT AUTISM SEVERITY CAN CHANGE DURING DEVELOPMENT?

An autism assessment provides a snapshot of the individual at a specific moment in time. A person’s autism presentation, however, is not constant across the lifespan. Just as people develop in every area of functioning, so does their autism change and develop with time. Studies evaluating this question have shown that for a substantial number of individuals, autism symptoms can significantly change in severity (Elias & Lord, 2022; Waizbard-Bartov & Miller, 2023). The percentage of autistic individuals showing change depends, in part, on which cohort of autistic individuals are described. More recent studies tend to show higher percentages of individuals demonstrating severity change. The tendency for change, however, differs among individuals; some decrease while others increase in severity. There is also change within individuals across different periods of their life; some decrease in severity during early childhood but increase in school years. Moreover, in the same way that developmental characteristics and co-occurring conditions impact autism presentation at a given period, they also affect its change over time.

Estimates of change in the severity of autism symptoms over time range from 11% to 58% depending on the cohort evaluated and the measures used (Waizbard-Bartov & Miller, 2023). Cohorts identified some time ago (such as the early-diagnosis sample) tend to report lower percentages of change (Gotham et al., 2012). More recently evaluated cohorts (such as the Pathways in ASD sample and the Autism Phenome Project sample) observe higher proportions of individuals that change in symptom severity (Georgiades et al., 2021; Waizbard-Bartov et al., 2022). Decrease in symptom severity ranges from 7% to 29% across different cohorts (Georgiades et al., 2021; Pellicano et al., 2019; Szatmari et al., 2015; Venker et al., 2014; Waizbard-Bartov et al., 2020).

The tendency to decrease in severity, however, is not uniform across development. Severity decrease tends to be more common during the preschool years compared to later in childhood (Fountain et al., 2012; Lord, Luyster, et al., 2012), when, at school-age, it can either decrease (Waizbard-Bartov et al., 2022), plateau (Georgiades et al., 2021) or increase in severity (Clark et al., 2017). Sex differences also exist, with young autistic girls showing a higher likelihood of reductions in severity than young autistic boys (Szatmari et al., 2015; Waizbard-Bartov et al., 2020, 2022). Conversely, between 8% and 29% of autistic individuals increase in autism symptom severity over time (Gotham et al., 2012; Kim et al., 2016; Pellicano et al., 2019; Waizbard-Bartov et al., 2020). It is not yet clear why some individuals increase in symptom severity across development while others do not. It has been repeatedly found that groups of autistic children increase in severity from an initially low average severity level at the beginning of early childhood (Gotham et al., 2012; Kim et al., 2016; Venker et al., 2014; Waizbard-Bartov et al., 2020). But, for individuals, levels of autism severity at a young age on their own are not strong predictors of future change, highlighting the crucial need for further investigation into this issue.

## WHAT INFLUENCES WHETHER AUTISM SEVERITY CHANGES OVER TIME?

Different factors can potentially impact change in core symptom severity levels. Many of these include developmental characteristics as well as the presence of co-occurring conditions. The fact that decrease in severity, especially in social-communication symptoms, is more common during younger ages suggests that reduction in symptoms involves rapid development of language, which tends to occur during the preschool years (Bal et al., 2019). Indeed, children who experience speech delays or remain minimally verbal are less likely to decrease in severity during early childhood (Bal et al., 2019). IQs within the normal range also characterize individuals who decrease in autism symptoms (Georgiades et al., 2021; Gotham et al., 2012; Waizbard-Bartov et al., 2020; Woodman et al., 2015). Conversely, autistic children around age 2 who were functioning below the developmental age of 12 months showed minimal developmental gains in the next 2 years, especially in the area of social communication (Hinnebusch et al., 2017). Cognitive ability is associated with other lifestyle factors that can influence changes in autism symptom severity. For example, children with typical range IQ are more likely to attend inclusive schools. By doing so, they are exposed to neurotypical peers who can serve as models for complex social interactions and learning experiences (Pellicano, 2012; Simonoff et al., 2019; Woodman et al., 2016). In fact, those individuals that “lose” their autism diagnosis, (i.e., decrease in symptom severity to no longer meeting the diagnostic criteria for autism—now referred to as Loss of Autism Diagnosis (LAD), Eigsti et al. (2022)), tend to have typical-range IQ and attend inclusive educational settings (Elias & Lord, 2022; Fein et al., 2013; Lord & Jones, 2012). In some studies, these children with LAD, also had significantly more behavioral therapy between ages 2 and 3 (Orinstein et al., 2014) and showed a reduction in RRBs over the same period (Anderson et al. (2014)). Moreover, having relatively strong cognitive skills can also support a child’s ability to engage in intervention, and to practice and generalize the tools received (e.g., social skills) in real-life settings (Hudry et al., 2018). Children with intellectual disability, in contrast, often attend special education settings and are not afforded the same exposure to neurotypical environments though they may receive other kinds of needed support. Finally, facets of the social psychology of being a girl might convey specific advantages for symptom severity decrease. Girls’ environments tend to put a higher emphasis on, and present more opportunities for, social interaction compared to boys’ (Bargiela et al., 2016; Dean et al., 2017), serving as inherent learning opportunities for social and communication skills and perhaps experiencing higher expectations from others. It is important to note, however, that attempting to meet such social demands might also lead autistic females to “camouflage,” that is, mask their autism symptoms in social settings (Hull et al., 2017), which can be experienced as stressful and can lead to detrimental effects for mental health (Hull et al., 2021). It is also possible that biological differences between boys and girls that contribute to sex differences in typical children also contribute to social symptom decrease in autistic girls, but this has not yet been rigorously investigated.

Often, individuals who increase in symptom severity also increase in other types of mental health problems (Baribeau et al., 2022; Waizbard-Bartov et al., n.d.; unpublished observations). Conversely, individuals who show marked decreases in symptom severity may also show decreases in comorbid psychopathology (e.g., anxiety, depression, oppositional defiant disorder; Orinstein et al. (2015)). These parallel increases and decreases make it hard to tease apart behaviors resulting from autism symptoms from those resulting from other psychopathology. For instance, both social-communication deficits as well as anxiety and depression can lead to impaired social functioning and social withdrawal (Duvekot et al., 2018; Hunsche et al., 2022). This makes it difficult to distinguish which condition is the cause for behaviors such as social isolation and avoidance. Another example is that having co-occurring anxiety is associated with higher levels of RRBs (Cashin & Yorke, 2018; Kim et al., 2020; Rodgers et al., 2012). Furthermore, RRBs and anxiety can also impact each other’s trajectories across development (Waizbard-Bartov et al., n.d.; unpublished observations). When experiencing distress, autistic individuals may use some forms of RRBs in order to regulate their anxiety level (Jaffey & Ashwin, 2022). Sensory sensitivities, a type of RRBs, can also lead to specific phobias focused on that sensory stimulus (Green & Ben-Sasson, 2010). Regardless of the diagnosis driving it, behaviors such as social withdrawal and avoidance (of people or stimuli) illustrate how the challenges associated with autism can impact a person’s functioning and well-being in daily life. Socioeconomic factors might also have an influence on whether a child increases or decreases their autism symptoms. Thus, living in an impoverished neighborhood (Simonoff et al., 2019) or having more poorly educated parents (Fountain et al., 2012; Waizbard-Bartov et al., 2022) are associated with increases in autism severity over time. Lai and Baron-Cohen (2015) discuss the theoretical and clinical difficulties in distinguishing true co-morbidities, overlapping symptoms, and differential diagnosis when considering ASD symptoms in relation to Obsessive-compulsive disorder (OCD), anxiety, depression and other conditions.

## WHAT IS THE LIMITATION IN RESEARCH EVALUATIONS OF AUTISTIC SEVERITY IN DETERMINING FUNCTIONAL IMPAIRMENT?

While we have indicated that autism symptom severity, and how it changes developmentally, is important and can be reliably measured, we now turn to the point that autism severity does not provide a complete understanding of the ramifications on quality of life of having autism. Perhaps the major reason that this is true is that the majority of individuals with autism are also diagnosed with other, co-occurring conditions (Lai et al., 2019) such as intellectual disability, language delays, sleep disorders, gastrointestinal symptoms, anxiety, depression, aggression, and so forth. The DSM definition of autism severity and the ADOS CSS, were not designed to measure autism severity in relation to these co-occurring characteristics. Rather, they were intentionally designed to evaluate autism symptoms *independent* of them (though not perfectly, see Gotham et al., 2009). But, in the context of functional outcomes and well-being, these co-occurring conditions can greatly impact the way the core symptoms are manifest in an individual’s behavior, as well as the extent to which autism impacts functioning in everyday life.

Consider, for example, an individual that received an ADOS CSS of 9 and a second individual that received an ADOS CSS of 5. The first individual would have showed more core-symptom-like behaviors during their ADOS assessment than the second. Comparing specific behaviors, it could mean, for example, that the first individual did not engage in any reciprocal interaction with the assessor, while the second did engage in conversation, but one characterized by poor “back and forth” quality. Similarly, the first individual (ADOS CSS 9) may have played during the ADOS but in a restricted, inflexible way, while the second individual (ADOS CSS 5) played in a more flexible manner that also included the assessor as an active participant in the play. Their behaviors during the ADOS session determined their severity level. But, what if the individual with ADOS CSS of 9 also had average IQ and fluent language, while the second individual, with ADOS CSS of 5, had cognitive disability and minimal speech? Wouldn’t we expect that such differences would affect the degree to which their autism impacts their everyday functioning in real life? In addition, emotional and behavioral issues, such as anxiety or agitation, that appear during the ADOS do affect the CSS and the same issues as reported in standardized measures are related to higher scores on caregiver-report autism measures such as the ADI-R or SRS (Havdahl et al., 2017). These differences, however, are not captured under the research definition of autism severity, based on core symptoms alone. This issue has been more exhaustively discussed in Pickles et al. (2020).

## HOW DOES HAVING AUTISM IMPACT A PERSON’S REAL LIFE?

Defining and evaluating autism severity based solely on the presentation of core symptoms has the benefit of being specific and measurable. Yet, it does not consider many other meaningful aspects of having autism and thus does not provide a full picture of the challenges and strengths faced by autistic individuals. To understand how autism impacts a person’s functioning, well-being and everyday life, we must understand how the different aspects of autism interact with each other at specific periods and across development. This cascading trajectory determines the way having autism manifests in a person’s life over time, impacting both objective functioning (i.e., employment, independence skills) as well as a subjective sense of wellbeing and quality of life. This suggests that it is important to evaluate how having autism impacts a person’s life in a more encompassing way, one that addresses the core symptoms as well as other influential aspects of an autistic person’s life. Such a multidimensional outlook could prove significant for clinical work, that is, identifying needs, planning intervention, assigning support, and creating future goals.

One imperfect but important type of measure for understanding an individual’s level of everyday functioning (Kanne et al., 2011) is a measure that places adaptive skills in communication, socialization, self-care, and motor abilities on an absolute age equivalence scale as well as a standardized score relative to age-matched peers. Examples of such instruments are the Vineland Adaptive Behavior Scale (Sparrow et al., 2017), the Adaptive Behavior Assessment System (Harrison & Oakland, 2015), and the Inventory for Client and Agency Planning (Bruininks et al., 1986), all frequently used with autistic individuals from very early childhood to adulthood (Losada-Puente & Bana, 2022). Several studies find that adaptive skills as measured on these instruments may be more highly correlated with cognitive functioning than with autism symptom severity and that adaptive skills tend to be lower than might be predicted by IQ in autistic individuals (Kanne et al., 2011; Weitlauf et al., 2014).

Another approach to assessing an individual’s real-life challenges is by using instruments focused on support needs in everyday life. These include, for example, the Support Intensity Scale (Thompson et al., 2004) and the Instrument for the Classification and Assessment of Support Needs (Arnold et al., 2009). Both of these tools provide a standardized approach to support needs by measuring, profiling and describing the types and amount of support needed for an individual to successfully engage in daily activities. In addition to objectively measured adaptive skills and support needs, intrapsychic well-being and quality of life are equally important in understanding how autism impacts a person’s everyday life. This calls for the development and use of measures of mood, self-esteem, and life satisfaction for individuals with autism and different levels of cognitive ability. Particular attention should be paid to developmental periods of physical and social transition, such as transition into adolescence and into early adulthood, when coping skills may need to be modified and when social demands and physical changes may place moods under particular stress. Core symptom severity levels have been repeatedly shown to influence quality of life: having higher or more severe autistic traits is associated with lower quality of life for adults (Capp et al., 2022), children and adolescents (Oakley et al., 2021) and preschoolers (as reported by their caregivers) (Lopez-Espejo et al., 2021). However, other factors related to having autism, particularly associated mental health conditions such as anxiety and depression, have also been shown to impact quality of life, even after accounting for core autism traits (Oakley et al., 2021).

There is a multitude of factors and dimensions along which the implications of having autism can be evaluated for impact on an individual’s life. The long-term goals proposed by McCauley et al. (2020) of *autonomy, daily living skills, relationships and employment/activities outside the home*, in forms that are consistent with the individual’s abilities and interests, are an excellent start for assessing outcomes. Every individual on the spectrum is first and foremost unique, characterized by a distinct profile of capabilities, challenges, desires and needs. These dimensions, however, can also be useful for defining subgroups of autistic individuals with common profiles concerning these dimensions and in terms of outcomes that could be expected, given their abilities and desires. One such subgroup was recently defined as “profound autism.”

## THE BENEFITS OF THE TERM PROFOUND AUTISM AND OF DEFINING DIFFERENT SUBTYPES OF AUTISM

*The Lancet Commission on the future of care and clinical research in autism* (Lord et al., 2022) provided a comprehensive overview of current diagnostic and intervention practices for ASD. Among many proposals to improve the lives of autistic individuals in the next 5 years, was the suggestion for the use of the term “profound autism.” This term highlights a clinical presentation of autism that includes high severity of core symptoms, co-occurring intellectual disability, little or no language and requiring extensive long-term care. For these individuals (and their caretakers), the challenges brought on by having autism are substantial and go well beyond the core characteristics. Having profound autism is impairing to functioning and independence and greatly impacts outcomes. A major advantage of this term is that it integrates both core and co-occurring conditions to represent the real-life challenges of an individual. Caution must be used, however, not to view individuals with profound autism as inferior or less deserving of their needed supports, due to the high severity of their challenges and disability compared to others on the spectrum. While there are reasonable concerns that providing a term associated with the greatest needs may stigmatize a group, almost all terms, no matter how gentle can be used to stigmatize. The onus is on us to challenge this stigmatization in whatever form it occurs.

The term profound autism, however, pertains to only a subgroup of individuals on the autism spectrum. Other individuals, those, for example, with intact cognitive and language abilities, have the potential for needing reduced, specific supports while leading independent lives. Recent work evaluating outcomes for 232 late-diagnosed autistic adults without intellectual disability in Germany reported that 50.4% had acquired university-entrance qualifications, 21.6% graduated from university and 74.8% were employed (Espeloer et al., 2022). Most importantly, findings from the EU-AIMS study suggest that 36%–71% of autistic individuals *do not* experience a reduced quality of life (Oakley et al., 2021). Such strengths do not imply that these individuals completely escape the struggles and challenges due to having autism. What might also characterize individuals in this group are well-developed coping skills that support their resilience. Skills such as self-advocacy (Kapp, 2020), the ability to compensate for symptoms in social settings (Livingston & Happe, 2017), creating strategies to restrict debilitating aspects of autism, for example, sensory overload (Clement et al., 2022) can all help to mitigate the challenges of autism in everyday life. Living in beneficial environments is also a strength that characterizes some individuals on the spectrum. This can be done, for example, by finding a peer group with similar interests, creating social connections that suit them (Tesfaye et al., 2022) and seeking out social and emotional support (Ghanouni & Quirke, 2022). Another example for establishing beneficial environments is engaging in employment that is suited to their own unique skills and abilities (Cheriyan et al., 2021), for instance, having abundant knowledge about specific topic areas. Some autistic individuals show impressive strengths *associated with* their autism, in areas such as memory, computation, music, visual learning (Bal et al., 2022), attention to details, and the ability to understand reasoning rules and systemizing (Baron-Cohen et al., 2009). For this group of individuals, their autism leads to a complex mixture of struggles and strengths, both of which are relevant when evaluating autism severity and how it impacts impairment in daily life. It would seem valuable to assign a specific name to this portion of the autism spectrum although members of this group might be the best to establish a self-referential terminology. At one point, the term “Asperger syndrome” was closely associated with this group though it was excluded from the latest Diagnostic and Statistical Manual because its use was so inconsistent and the opposite of stigmatization occurred in which families and individuals sought out an Asperger’s diagnosis because it sounded superior to autism, sometimes to find that they were then excluded from services because the diagnosis implied fewer needs (Lord & Jones, 2012; Lord, Petkova, et al., 2012).

The two subgroups described above represent two extreme ends of a highly heterogenous spectrum of autistic individuals who differ not only on autism severity but also concerning their needs, challenges and abilities. It would appear that most autistic individuals are somewhere in between these two extremes. These individual differences are what makes defining the way autism impacts people’s lives so challenging: it ranges widely, from being severely impairing to promoting a diverse, enriching sense of identity (Cooper et al., 2021). Using this overarching perspective on autism, other clinically meaningful subgroups could be identified, with the goal of encouraging each group to reach its fullest potential in adaptive functioning and subjective wellbeing and to generally flourish in life.

The two defined subgroups discussed above differ on many dimensions. In order to be able to classify all of the intermediate individuals, an attempt could be made to identify the most useful dimensions for both clinical characterization and research progress. Attempts at classifying autism subtypes have been many and varied; perhaps the first attempt at subgrouping by social functioning was as early as 1979 (Wing & Gould, 1979), classifying autistic children’s social functioning as Active, Passive, and Active but Odd.

Using a genetic approach, (Zhou et al., 2022) recently identified five new risk alleles in the very large SPARK database of autistic volunteers’ DNA. Unlike many other genes associated with autism, these new risk alleles do not appear to cause profound autism with intellectual disability. The authors suggest that there are many more single genes of large effect that can cause severe autism that remain to be discovered, but also that additional risk alleles will be discovered in very large samples, that confer a moderate likelihood of autism with intact cognition, potentially with higher intellectual, adaptive, and outcome status. Such approaches, using genetics, neuroanatomy, or neurophysiology as well as behavioral response to intervention and co-occurring conditions, may allow progress in accurate subgrouping of autistic individuals. However, Rapin (2014) discusses the three levels of understanding a developmental disorder like autism (genetic or environmental etiology, pathophysiology, and phenomenology) and cautions that relating categories or dimensions on one level to categories or dimensions on another has not been very successful to date, although it needs to be a goal for the future. The more attainable goal for the near future may be a more universally accepted dimensional characterization of behavior.

A promising model of such characterization was proposed for individuals with intellectual disability (Schalock & Luckasson, 2015). These dimensions are (a) intelligence, (b) adaptive behavior, (c) health, (d) participation in social activities, and (e) the personal and environmental context in which individuals live their daily lives. For autism, obviously, severity of social and RRB symptoms would have to be added. Fortunately, severity of autism symptoms, intelligence (verbal and nonverbal), and adaptive behavior already have available operationalized, standardized measures. Description of health issues for the autism population would probably benefit from two divisions: conditions that are primarily physical (but may have psychological consequences) such as epilepsy and GI disorders, and conditions that are primarily psychological (e.g., anxiety, depression, and obsessionality). Schalock and Luckasson (2015) suggest that their classification system of ID could serve four purposes: describing functional levels, operationalizing the level of supports needs, defining health status, and determining legal status, in relation to specific areas and contexts. Such purposes are very relevant and appropriate for the clinical needs of the autism population, and may promote accurate research into statistically determined subtypes, type and intensity of supports needed, and outcomes of intervention research, although basic biological research might have to involve more finely detailed characterization (Waterhouse and Gillberg’s “micro-groups” (2014)).

## ENVIRONMENTAL INFLUENCES ON AUTISM SEVERITY

It will be obvious that the dimensions proposed by Schalock and Luckasson (2015), plus autism severity, will not be independently developing domains. Beyond the severity of core symptoms and the occurrence of co-occurring health or intellectual conditions, the environment plays a meaningful role in the way autism impacts a person’s life. Having access to more resources in the parental and home environment (Fountain et al., 2012; Simonoff et al., 2019; Waizbard-Bartov et al., 2022) as well as to early diagnosis (Gabbay-Dizdar et al., 2021) and intervention (Pickles et al., 2016) can help promote gains and mitigate impairments over time. Moreover, the environment in which a person lives is not static, but rather changes with time. Some aspects of autism can have differential impacts, meaning they are impairing to different degrees during various developmental stages, depending on the challenges, demands and support available during that stage (Bal et al., 2019). For example, increased social complexities along with decreased resources and support characterize the transition from adolescence to young adulthood, potentially leading the same level of autistic symptoms to have more impairing outcomes for everyday life (Taylor & Seltzer, 2010). With age, enhancing person-environment fit can contribute to an individual’s resilience, in addition to promoting individual skills (Lai & Szatmari, 2019). There are different ways in which environments can support better fit. Providing adequate services across the duration of development is one example (Laxman et al., 2019). Creating opportunities to engage with non-autistic peers, such as growing up with neurotypical siblings or attending inclusive educational settings (Pellicano, 2012; Woodman et al., 2016) are naturalistic learning opportunities for social modeling and to practice social interaction. On the other hand, autistic individuals and stakeholders repeatedly indicate how environmental stressors such as social biases, negative attitudes, and stigmatization toward people with autism lead to detrimental outcomes and play a major role in their real-life challenges (Cohen et al., 2022; Ghanouni & Quirke, 2022).

## CONCLUSIONS AND FUTURE DIRECTIONS

The current definition of autism severity, and the way it is measured in research, is based solely on the severity levels of the two core symptom domains, controlling for age and language level. The challenges faced by autistic individuals in real life, however, go far beyond core symptoms. Common, co-occurring conditions such as intellectual disability, language delays, and anxiety disorders are as impairing to functioning and wellbeing for many individuals as are the core symptoms themselves. Moreover, core symptoms and co-occurring conditions can interact across development, each influencing the other’s trajectory over time. Translating this multidimensional outlook into a measurable, formal definition, that captures the combined influences of having autism on a person’s life, as well as providing a detailed characterization for research, presents an unfulfilled challenge. The multidimensional approach promoted for ID (Schalock & Luckasson, 2015), encompasses the impact of IQ scores, but adds other important factors, including adaptive behavior and support needs. In the DSM-5, the severity levels for the core symptoms of autism are already posited to correspond to levels of functional impairment. This further supports the idea that in autism, as is the case for intellectual disability, characterizing autistic individuals for both clinical and research purposes should include not only severity level but should take into account other impactful dimensions that are part of the condition and influence people’s everyday life. If created, a multidimensional, measurable definition of autism severity could potentially be useful for identifying unique subgroups of individuals for clinical purposes, for determining individual needs and strengths in clinical assessments, and for developing intervention goals and plans that involve all the different aspects and challenges relevant to the life of a person with autism. Fein and Helt (2017) also suggest several approaches that could be included in classifying research “micro-groups” (Waterhouse & Gillberg, 2014), such as noting behaviors that seem relatively impervious to environmental differences (e.g., high pain thresholds, social improvement with fever), studying emergence of autism in the first 2 years of life, before intervention has started, and including longitudinal course as a classifier (improving, worsening, and response to intervention, as described above).

One caution is that dimensions and specific variables that are used to characterize individuals with autism are not necessarily the best “outcome” variables. In many cases, baseline characterization will serve to describe the individual at one point in time, while outcome variables may assess change in that variable over time or because of an intervention.

Given this caution, however, one promising avenue in this regard is the development of a core outcome set (COS) for autism. A COS identifies the domains of a condition that are most relevant to clinicians, caregivers and individuals with autism. Recently, a COS for autism was developed by The International Consortium of Health Outcome Measurement in an attempt to calibrate severity along multiple dimensions and with different assessment tools (Patient-centered outcome mesasures, 2022). This system lists published instruments that can characterize core symptoms (social communication and RRBs), as well as adaptive skills, family functioning, sleep and anxiety, other neurodevelopmental disorders (as measured by the Child Behavior Checklist) and general quality of life. This is one promising attempt to conceptualize autism severity based on different components of the clinical presentation, authored primarily by clinicians and researchers with expertise in autism phenomenology. Hopefully, others will take up this system which will allow researchers and clinicians to have a comprehensive characterization of the individual, as well as to select dimensions that are most relevant to their basic biological and intervention studies.

## Data Availability

Data sharing is not applicable to this article as no new data were created or analyzed in this study.
